# T196. CALRETININ INTERNEURON DENSITY IN THE CAUDATE NUCLEUS IS LOWER IN SCHIZOPHRENIA

**DOI:** 10.1093/schbul/sby016.472

**Published:** 2018-04-01

**Authors:** Istvan Adorjan, Bin Sun, Virginia Feher, Teadora Tyler, Bori Damo-Csorba, Benedek Pour, Daniel Veres, Olaf Ansorge, Steven Andrew Chance, Francis Szele

**Affiliations:** 1 Semmelweis University; 2 University of Oxford

## Abstract

**Background:**

The excitatory/inhibitory imbalance theory is widely accepted in the pathology of autism spectrum disorder. Recent results suggest its relevance in the aetiology of schizophrenia as well (Jardri 2016, Yang 2017, Gao and Penzes 2015). In order to discover the possibly altered neuronal composition in schizophrenia numerous studies have been focussing mainly on different cortical regions such as the ventromedial prefrontal cortex and dorsolateral prefrontal cortex. In particular, various interneuronal populations have been found altered.2 However, relatively little is known about the neuroanatomical changes of subcortical structures, such as the caudate nucleus, in the pathology of schizophrenia.

**Methods:**

Therefore, we examined the immunohistochemical distribution of calretinin (CR) and NPY-immunopositive neurons in the caudate nucleus and the dorsolateral prefrontal cortex. The state of microglial activation was controlled by the detection of Iba1 and TMEM119. In order to corroborate our results obtained by immunohistochemistry (IHC) qPCR analyses were also conducted.

**Results:**

The present study provides evidence for the altered interneuronal composition of caudate nucleus in schizophrenia without signs of microglial activation. There were small, medium and large CR-immunopositive (CR-ip) interneurons detected in the caudate nucleus. There was a 32% decrease in the density of all CR-ip interneurons (p=0.020, statistical power=0.747) that was driven by the loss of the small CR-ip interneurons (p=0.017, statistical power=0.777) while the densities of the medium and large CR-ip and NPY-ip interneurons were not significantly altered (p=0.078, p=0.436, p=0.125, respectively). Our experiments were also extended to the dorsolateral prefrontal cortex (medial frontal gyrus and superior frontal gyrus) where no significant changes were seen by IHC. However, qPCR analyses revealed a trend of decreased CR mRNA levels in schizophrenia (p=0.061, statistical power=0.485) while returned no significant changes regarding mRNA levels of NPY, Iba1 and TMEM119. No significant interactions between variables were seen controlling for PMI, age and gender by univariate, multifactorial ANOVA.

**Discussion:**

We have discovered one of the most striking examples of altered neuronal densities in the forebrain in schizophrenia; a highly significant decrease in CR-ip neuronal density in the caudate nucleus. Future studies are warranted to elucidate neuronal inputs to CR-ip neurons in the caudate nucleus and whether they innervate local interneurons or medium spiny neurons. If they primarily regulate local interneurons they may disinhibit medium spiny neurons. If they directly innervate medium spiny neurons they may inhibit the principal cells of the striatum. It will also be important to determine if the large, medium and small CR-ip neurons have different connectivity and thus segregate function. Interestingly our results regarding the decreased density of CR-ip interneurons are in line with our previous observations in ASD1 that underline the possible shared pathomechanisms between schizophrenia and ASD.

**References:**

1. Adorjan I, Ahmed B, Feher V, Torso M, Krug K, Esiri M, Chance SA, Szele FG. 2017. Calretinin interneuron density in the caudate is lower in autism spectrum disorder. Brain 140:2028–2040.

2. Brisch R et al. 2015. Calretinin and parvalbumin in schizophrenia and affective disorders: a mini-review, a perspective on the evolutionary role of calretinin in schizophrenia, and a preliminary post-mortem study of calretinin in the septal nuclei. Frontiers in Cellular Neuroscience 9:393.

